# Ecological Perspectives on Leishmaniasis Parasitism Patterns: Evidence of Possible Alternative Vectors for *Leishmania* (*Leishmania*) *infantum* (syn. *L. chagasi*) and *Leishmania* (*Viannia*) *braziliensis* in Piauí, Brazil

**DOI:** 10.3390/pathogens14090930

**Published:** 2025-09-16

**Authors:** Raimundo Leoberto Torres de Sousa, Thais Araujo-Pereira, Silvia Alcântara Vasconcelos, Simone Mousinho Freire, Oriana Bezerra Lima, Jacenir Reis dos Santos-Mallet, Mauricío Luiz Vilela, Victor Manoel de Sousa Vasconcelos, Etielle Barroso de Andrade, Régis Gomes, Clarissa Teixeira, Bruno Moreira Carvalho, Daniela Pita-Pereira, Constança Britto

**Affiliations:** 1 Laboratório de Biologia Molecular e Doenças Endêmicas—LABIMDOE, Instituto Oswaldo Cruz, Fundação Oswaldo Cruz, Rio de Janeiro 21040-360, Brazil; leoberto.torres@gmail.com (R.L.T.d.S.); tap@ioc.fiocruz.br (T.A.-P.); danypyta@gmail.com (D.P.-P.); 2 Laboratório de Vigilância Entomológica—LVE Fundação Oswaldo Cruz, Teresina 64051-110, Brazil; silviaav@live.com (S.A.V.); jacenir.mallet@fiocruz.br (J.R.d.S.-M.); mvilela.fiocruz@gmail.com (M.L.V.); 3 Laboratório de Zoologia e Biologia Parasitária—ZOOBP, Universidade Estadual do Piauí-UESPI, Teresina 64002-150, Brazil; simonemousinho@ccn.uespi.br; 4 Gerência de Zoonoses de Teresina—GEZOON, Teresina 31741-405, Brazil; orilimamestrado@gmail.com; 5 Laboratório de Vigilância e Biodiversidade em Saúde, Universidade Iguaçu, Nova Iguaçu 26260-045, Brazil; 6 Laboratório Interdisciplinar de Vigilância Entomológica em Díptera e Hemíptera—LIVEDIH, Instituto Oswaldo Cruz, Fundação Oswaldo Cruz, Rio de Janeiro 21040-360, Brazil; 7 Grupo de Pesquisa em Biodiversidade e Biotecnologia do Centro-Norte Piauiense—BIOTECPI, Instituto Federal do Piauí—IFPI, Pedro II 64255-000, Brazil; vmanoel0321@gmail.com (V.M.d.S.V.); etlandrade@ifpi.edu.br (E.B.d.A.); 8 Laboratório de Imunoparasitologia, Fundação Oswaldo Cruz, Eusébio 21040-900, Brazil; regis.gomes@fiocruz.br (R.G.); clarissa.teixeira@fiocruz.br (C.T.); 9 Barcelona Supercomputing Center, 08034 Barcelona, Spain; brunomc.eco@gmail.com; 10 Laboratório de Biologia Molecular, Centro Universitário Lusíada—UNILUS, Campus II, Santos 11045-101, Brazil

**Keywords:** Phlebotominae, disease vectors, host–parasite interactions, *Leishmania* spp., environmental monitoring, Piauí state

## Abstract

Leishmaniasis is difficult to control due to clinical and vector diversity associated with the complex life cycle of *Leishmania* parasites, which are transmitted by sandflies. This study investigated the presence of *Leishmania* DNA in sandfly vectors, their blood meal sources, and their distribution in relation to environmental and climatic variables in four municipalities in Piauí state, Brazil. Between 2020 and 2022, sandflies were collected, morphologically identified, and analyzed for the presence of parasite DNA and blood meal sources (PCR, sequencing). Climate data were correlated with the density of collected insects. Among the 10,245 specimens collected, *Lutzomyia longipalpis* (54.87%) and *Nyssomyia whitmani* (30.41%) were the most abundant in the collection areas. *Leishmania braziliensis* DNA was detected in *Lu. longipalpis*, while *L. braziliensis* and *Leishmania infantum* DNAs were recovered from *Ny. whitmani. Homo sapiens* was the main blood meal source (~73%). Vector density was associated with humidity, temperature, and precipitation in Teresina and Pedro II, with significant results for *Ny. whitmani*. In conclusion, *Lu. longipalpis*, widely adapted to anthropized environments, can act as a potential vector of the etiological agent of cutaneous leishmaniasis in Teresina and Oeiras. In Pedro II, the detection of *L. infantum* DNA in *Ny. whitmani* suggests a possible role of this species in the transmission cycle of visceral leishmaniasis, reinforcing the complex ecoepidemiology of *Leishmania* spp. in Piauí.

## 1. Introduction

Leishmaniasis, a parasitic infection caused by kinetoplastid protozoans of the genus *Leishmania*, transmitted by sandflies (Diptera: Psychodidae), is a matter of great concern due to its global distribution and impact on public health worldwide [1]. The complex composition of transmission cycles of *Leishmania* parasites results in three main clinical presentations of the disease: visceral leishmaniasis (VL), cutaneous leishmaniasis (CL), and mucocutaneous leishmaniasis (ML). The prevalence of the different clinical forms is directly influenced by the species-specific interaction between the transmitted parasite and the sandfly vector [1,2,3,4].

However, studies have revealed that permissive phlebotomine sandfly species can be infected by a variety of *Leishmania* parasites, thus increasing the range of potential vectors [4]. For example, *Lutzomyia longipalpis* (Lutz & Neiva, 1912), a known vector of *Leishmania* (*Leishmania*) *infantum* (syn. *L. chagasi*), the etiological agent of VL in Latin America, has recently been found to be infected with *Leishmania* (*Leishmania*) *amazonensis*, which causes ML in Brazil [5]. This finding challenges the paradigm established by Killick-Kendrick (1990) [6] concerning the criteria to incriminate a species as a competent vector, and points to the need for further research on the ability of previously unconsidered vectors to transmit *Leishmania* parasites involved with the distinct clinical presentations of the disease.

The increase in leishmaniasis cases over the years has been attributed, in part, to the unplanned expansion of urban areas. This process alters the environment, creating favorable conditions for the propagation of phlebotomine sandflies, increasing human and animal contact with the vector [7,8,9,10]. Studies have reported additional vector species involved in the transmission of the causative agent of VL, including, but not limited to, *Pintomyia fischeri* (Pinto, 1926), *Migonemyia migonei* (França, 1920), and *Nyssomyia neivai* (Pinto, 1926) [11,12,13]. The presence of these vectors in urban areas represents a significant public health concern, highlighting the urgency of further research and the importance of entomological surveillance by authorities.

Piauí, a state located in the northeastern region of Brazil, presents a distinct ecological setting that influences the spatial distribution patterns of phlebotomine sandflies [14]. Several species have already been identified within this state, including *Nyssomyia whitmani*, recognized for its role in the transmission of *L. braziliensis*, the main causative agent of CL in the country. The presence of *Ny. whitmani* in Piauí, along with *Lu. longipalpis* (the remarkable vector of *L. infantum*) in urban, peri-urban and rural areas, suggests that the dynamics of *Leishmania* parasite transmission in the state may be affected by anthropogenic environmental changes, emphasizing the complexity of controlling the disease [14,15,16,17].

When analyzing the epidemiology of leishmaniasis in Piauí from an environmental perspective, the transmission dynamics appear to be strongly influenced by ecological aspects, particularly in anthropized environments [18]. The expansion of urban areas has altered the natural environment of sandflies, favoring their proliferation and consequently impacting the transmission cycles [9,19,20].

Biological, ecological and evolutionary criteria determine the spatial distribution of vector species, which is affected by variables including climate and geographical space [15]. Species distribution is characterized by a hierarchical structure, with vectors demonstrating adaptability to the abiotic factors present in their specific environment. Climate, in particular, plays a crucial role in shaping the spatial arrangements of each species, from the microenvironment to global scales [15].

In addition, sandfly feeding patterns are crucial for facilitating parasite transmission. Female sandflies, in particular, feed on hemoglobin-rich blood from humans as well as on domestic and wild animals [21,22]. The range of available vertebrate hosts in the environment favors sandfly adaptation to urban areas and may have an impact on their vectorial competence, thereby affecting parasite transmission [23,24].

In this context, the aim of the study was to investigate the presence of *Leishmania* spp. DNA in phlebotomine sandflies to assess their potential role in the transmission cycles of parasites causing cutaneous and visceral leishmaniasis, as well as to identify the feeding sources of these insects. Beyond that, the study examined possible correlations between sandfly density and both environmental and climatic factors across four selected municipalities in Piauí, distributed in different biomes and presenting diverse climatic typologies.

## 2. Materials and Methods

### 2.1. Study Area

The state of Piauí, located in northeastern Brazil (5°05′25.2″ S; 42°48′40.1″ W), is divided into 224 municipalities, 19 immediate geographic regions, and 6 intermediate geographic regions [25,26]. Piauí presents a land area of 251,755 km^2^, an estimated population of 3,289,290 inhabitants, and a population density of 12.40 individuals/km^2^ [25]. The state has three types of regional climatic diversity: tropical savannah, hot semi-arid, and hot desert climates [27]. The vegetation is also diverse, consisting of caatinga, cerrado, deciduous forest, and palm forest.

**Figure 1:**
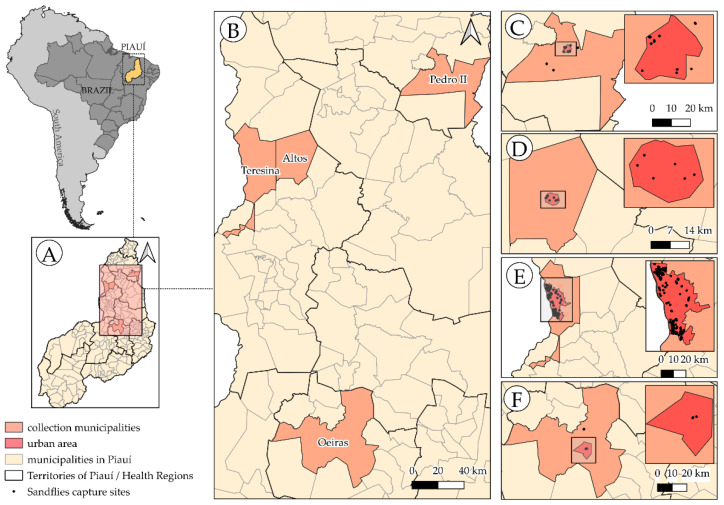
Map of Piauí highlighting the four municipalities selected for sandfly collection, their urban areas, and their respective collection sites: (**A**) The state of Piauí, with its divisions into municipalities and territories. (**B**) Municipalities where sandflies were collected. (**C**) Pedro II. (**D**) Altos. (**E**) Teresina. (**F**) Oeiras. Black dots represent the collection sites. Software used: QGIS Version 3.40.4-Bratislava; Shapefile Source: IBGE (Brazilian Institute of Geography and Statistics). Available at https://www.ibge.gov.br/geociencias/organizacao-do-territorio/malhas-territoriais/15774-malhas.html?edicao=36516&t=acesso-ao-produto (accessed on 17 March 2023).

The municipalities studied are distributed across different biomes and climatic typologies, which can cause differences in the parasite transmission dynamics. The cities of Teresina, Altos, Pedro II, and Oeiras were selected due to an increased number of VL and ACL cases from 2013 to 2023 and by differences in vegetation type, climatic factors, and biomes among them. The selected areas and their respective collection sites are shown in Figure 1.

Teresina, the capital of Piauí, is located at coordinates (5°05′11.0″ S 42°48′11.0″ W), on the border with the state of Maranhão, at an altitude of 72 m above sea level. The city has an estimated population of 902,644 inhabitants and has two seasons: rainy, from November to March, and dry, from April to October [28,29].

Altos (5°02′22.9″ S 42°27′36.1″ W), a municipality located in the Teresina Metropolitan Region, has an estimated population of 49,637 inhabitants, with a population density of 49.57 individuals/km^2^. The city has climatic seasons similar to those of Teresina due to the proximity of the two cities [29,30].

Pedro II (4°25′34.9″ S 41°27′24.7″ W), in the north of Piauí, has an estimated population of 39,039 inhabitants with a population density of 24.54 individuals/km^2^. The municipality is positioned at an altitude of 603 m, reaching 700 m at its highest point, and is situated in a region of Piauí characterized by a mild climate. Because it is in the same climate region as Teresina and Altos (Aw-Tropical Savanna Climate), Pedro II also has two seasons: rainy, from November to March, and dry, from April to October [29,31].

Oeiras (7°00′59.3″ S 42°07′26.6″ W) has an estimated population of 39,545 inhabitants. Its territorial area is 2703 km^2^, with a population density of 14.12 individuals/km^2^. The climate is tropical semi-arid, with rainfall concentrated between December and April and a dry season between June and September [32] (Figure 1).

### 2.2. Sandfly Collection and Morphological Identification

Collections were conducted from November 2020 to August 2022. Collection sites were selected based on their environmental characteristics indicative of the establishment and maintenance of vector breeding sites, i.e., residences with extensive peridomicile areas, the presence of abundant vegetation, mainly shrubs, with accumulated organic matter in the soil, and the presence of domestic animals, which serve as probable food sources for sandflies.

Sandflies were collected using light traps (HP/CDC model) [33] placed either on the ground (50–100 cm high) or at treetop level (~10 m high) in the peridomiciliary areas. The traps were set up for 14 h (from 17:00 to 07:00 of the following day). Collections in Pedro II and Teresina were conducted over a 12-month period, whereas those performed in Oeiras and Altos were of shorter duration, from 3 to 6 months. In total, more than 6000 h of collection effort were accumulated in all the selected municipalities (Table S1).

After the collections, insects were transported alive to the laboratory and euthanized by freezing (−20 °C) for 40 min. They were then stored in 70% ethanol, and a preliminary sorting was performed to separate male and female sandflies from other insects. Phlebotomine sandflies were classified according to the collection sites in each municipality.

A second sorting separated non-engorged females for *Leishmania* spp. DNA detection, from engorged females, for blood meal source identification. To carry out the morphological identification of species, the head and the last three abdominal segments of all females were sectioned for microscopic observation of the genital tract and cephalic structures (palps and cibarium); the thorax and the rest of the abdomen were placed into 1.5 mL tubes and stored at −20 °C until DNA extraction.

The head and final segments extracted from females, together with all males, were clarified and diaphanized according to the method described by Forattini in 1973 [34]. After processing the material, the species were identified under an optical microscope, following the identification methodology of Galati established in 2019 (and updated in 2024) [35]. The proposal for abbreviations of genus and subgenus was drafted according to Marcondes et al.’s work from 2007 [36].

### 2.3. Analysis of Meteorological Data

Climatic conditions, including temperature and relative humidity, were recorded using a thermo-hygrometer, while rainfall data were obtained from the National Meteorological Institute (INMET) (https://portal.inmet.gov.br/ (accessed on 23 May 2023)). To visualize the annual temperature panorama, data extracted from the weatherspark website (https://pt.weatherspark.com/ (accessed on 23 May 2023)) were analyzed and organized into a heat chart for the four municipalities: Pedro II, Altos, Teresina, and Oeiras. These heat map graphs are color-coded by the website into four categories: “comfortable” (18 to 24 °C), “warm” (25 to 29 °C), “hot” (30 to 35 °C), and “sweltering” (above 35 °C) (Figure S1).

### 2.4. Relationship Between Altitude and Species of Sandflies

This study used a systematic approach to investigate the correlations between collection altitudes and the dispersal patterns of the two species of sandflies, *Lu. longipalpis* and *Ny. whitmani*. The R software version 4.2.2 was used, using the “terra” package to obtain altimetry data for each sampling site based on their geographical coordinates.

The selection of both species for analysis was based on their higher frequency compared with the other species collected, thus justifying their inclusion due to their epidemiological importance in Piauí. Subsequent procedures involved performing descriptive calculations on the “altitude” variable to determine the average, minimum, and maximum values of this altimetric attribute.

For a visual representation of data, boxplots were constructed showing the distribution of altitudes for the occurrence of *Lu. longipalpis* and *Ny. whitmani species*. The visualization was generated using the ggplot2 package in the R software [37].

### 2.5. Detection of Leishmania *spp.* DNA

The detection of *Leishmania* spp. DNA in sandfly females was conducted following previously described protocols of our group [38,39]. Initially, the non-engorged females were organized into pools with a maximum of 10 specimens, each pool consisting of individuals of the same species, municipality, and ecotope (peridomicile or intradomicile).

The biological material of each pool of non-engorged females was submitted to DNA extraction using the Wizard SV Genomic DNA Purification System (Promega, Madison, WI, USA), according to the manufacturer’s specifications. DNA samples were assayed in a multiplex PCR [40] able to simultaneously amplify the conserved minicircle region of *Leishmania* spp. kDNA (primer A: 5′ GGC CCA CTA TAT TAC ACC AAC CCC 3′; primer B: 5′ GGG GTA GGG GCG TTC TGC GAA 3′) [38] and the IVS6 region of the cacophony gene of neotropical sandflies (5Llcac: 5’ GTG GCC GAA CAT AAT GTT AG 3’; 3Llcac: 5’ CCA CGA ACA AGT TCA ACA TC 3’) [39]. The latter served as internal control for the enzyme activity (by checking for the presence of potential inhibitors in the insect samples), DNA yield, and purity [41]. Strict procedures were followed to prevent contamination. Male sandflies were included as negative controls for the DNA extraction step, and artificially infected females were added as positive controls in each PCR run [40].

### 2.6. Identification of Leishmania *spp.*

*Leishmania* species identification in the kDNA-positive sandfly pools was performed by PCR amplification of the Leishmania hsp70 gene (primers hsp70C forward: 5′ TCC TTC GAC GCC TCC TGG TTG 3′; hsp70C reverse: 5′ GGA CGA GAT CGA GCG CAT GGT 3′), generating a 234 bp fragment, as previously described [41]. The 234 bp amplified products were subjected to a second PCR round using the hsp70C reverse primer and a new one (hsp70F2 forward: 5′ GGA GAA CTA CGC GTA CTC GAT GAA G 3′) [42], giving rise to an internal 144 bp region of the hsp70 gene. The 144 bp products were cloned using pGEM^®^-T Easy Vector Systems (Promega, Madison, WI, USA), following the manufacturer’s standard procedures. The commercial PureLink Quick Plasmid DNA Miniprep kit (Invitrogen, Carlsbad, CA, USA) was used for DNA extraction of recombinant plasmids containing the *Leishmania* DNA insert of each positive pool, according to the manufacturer’s protocol, and the genetic material was submitted to sequencing.

### 2.7. Identification of Blood Food Sources of Engorged Females

Each engorged female was placed into individual tubes for food source identification by PCR and sequencing. DNA extraction from the thorax and abdomen of each specimen followed the same protocol used for the non-engorged sandfly pools. The source of blood meals was determined by PCR directed to the 12S rRNA gene of vertebrates (primers: forward 5′ CCC AAA CTG GGA TTA GAT ACC C 3′, reverse 5′ GTT TGC TGA AGA TGG CGG TA 3′) [43]. The 215 bp amplified fragment was eluted from 2% agarose gel, purified using the Illustra GFX PCR DNA and Gel Band Purification Kit (GE Healthcare, Pittsburgh, PA, USA), and submitted to sequencing. In the PCR assays, DNA extracted from male sandflies was used as a negative control, and DNA from *Lu. longipalpis* females experimentally fed on rabbit blood (inactivated at 56 °C) served as a positive control.

### 2.8. Sample Sequencing

For both *Leishmania* spp. identification (purified 144 bp hsp70 DNA insert) and food source analyses (purified 215 bp DNA fragment from the 12S rRNA gene), the genetic material was subjected to sequencing. Sequencing reactions were performed using Big Dye Terminator v.3.1 Cycle Sequencing (Applied Biosystems, Foster City, CA, USA), following the manufacturer’s specifications, and analyzed on the ABI 3730 Sanger DNA system at the Oswaldo Cruz Foundation (PDTIS/Fiocruz), Rio de Janeiro, Brazil. Generated sequences were analyzed and corrected, if necessary, using the Geneious software (V.10.1.3) [44]. Consensus sequences were then assembled for each sample and analyzed for similarity to other NCBI sequences using the BlastN tool [45] (http://www.ncbi.nlm.nih.gov/BLAST (accessed on 13 September 2023)).

### 2.9. Data Analysis

Chi-square tests (χ^2^) were used to compare male and female sandfly proportions with a 5% significance level. To analyze the relative abundance of collected sandfly species, to identify the most common species, and to investigate temporal and spatial variations, descriptive measures and statistical tests were applied. The minimum rate of positive sandflies for the presence of *Leishmania* DNA was calculated by multiplying the number of positive pools for each species by 100 and dividing by the total number of individuals of the respective species. In addition, the Kruskal–Wallis test was used to compare the number of sandflies between the surveyed municipalities. Spearman’s non-parametric correlations were applied to explore the relationship between climatic variables, altitude, and the presence of sandflies. The data were analyzed using R software, version 4.3.1.

### 2.10. Ethical Aspects

Collections were made under a license from the Biodiversity Authorization and Information System (SISBIO) for capturing zoological material (process 70965-5). Considering that this is an entomological survey and environmental analysis, with no involvement with vertebrate animals or organisms, the activity under analysis did not require submission to the Ethics Committee for research with human beings or animals. The obtention of *Lu. longipalpis* females experimentally fed on rabbit blood, as a positive control in the PCR assays for food source research, was approved by the Ethics Committee for Animal Use (process 23075.064300, license 1663).

## 3. Results

### 3.1. Faunal Density of Sandflies

In total, 10,245 sandflies were collected during the period of November 2020 to August 2022 in the municipalities of Teresina, Pedro II, Oeiras, and Altos in Piauí state; of these, 2591 were females and 7654 were males. The proportion of males (74.71%) was significantly higher than that of females (25.29%) [χ^2^ = 18.467; (df) = 4; *p* < 0.01], distributed over the four municipalities in an approximate ratio of three males for each female.

Table 1 describes the abundance and distribution of sandfly species in the four municipalities of Piauí. The identification of collected sandflies resulted in nine species belonging to the *Lutzomyia* sp., *Nyssomyia* sp., *Migonemyia* sp., *Evandromyia* sp., *Sciopemyia* sp., and *Brumptomyia* sp. genera, namely: *Lutzomyia longipalpis* (Lutz & Neiva, 1912) (5621, 54.87%), *Nyssomyia whitmani* (Antunes & Coutinho, 1939) (3116, 30.41%), *Migonemyia migonei* (França, 1920) (789, 7.70%), *Nyssomyia intermedia* (Lutz & Neiva, 1912) (521, 5.09%), *Evandromyia carmelinoi* Ryan, Fraiha, Lainson & Shaw, 1986 (132, 1.29%), *Sciopemyia sordellii* (Shannon & Del Ponte, 1927) (60, 0.59%), *Evandromyia lenti* (Mangabeira, 1938) (3, 0.03%), *Evandromyia termitophila* Martins, Falcão & Silva, 1964 (2, 0.02%), and *Brumptomyia avellari* Costa Lima, 1932 (1, 0.01%).

*Lu. longipalpis* was predominant, with an abundance rate of 54.87% of the total sandflies. On the other hand, Pedro II had the highest number of *Ny. whitmani* specimens, which in total represented 30.41% of the insects collected in all municipalities. The other species ranged from 0.01% to 7.70% across all surveyed localities (Table 1).

### 3.2. Climate Variables and Distribution of Sandflies

The study of the seasonal distribution of sandflies was conducted only in the municipalities of Teresina and Pedro II due to the large number of collected specimens in comparison with Oeiras and Altos.

**Figure 2:**
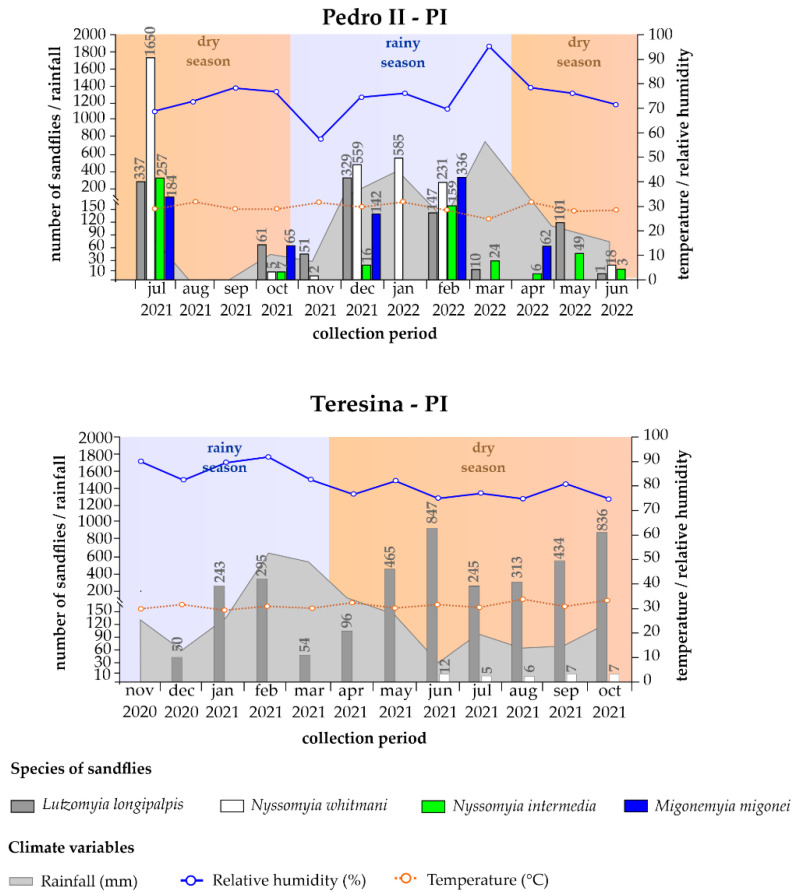
Climatic variables and sandfly density. Relationship between vector densities and rainfall (mm), temperature (°C), and relative humidity (%) from November 2020 to June 2022, in the municipalities of Pedro II and Teresina, state of Piauí (PI).

In Pedro II, there was a peak in the abundance of individuals of the species *Ny. whitmani* (1650/3050) observed during the dry season, in July 2021. During the rainy season (December 2021 to February 2022), the predominant species continued to be *Ny. whitmani*, which showed a decrease in density but remained higher than the other species, especially in December (559 individuals) and January (585 individuals). The species *Lu. longipalpis* showed a lower population during the rainy season, with peaks in December (329 individuals) and February (147 individuals). On the other hand, the species *Ny. intermedia* and *Mi. migonei* increased their population density during the rainy period, mainly in February, preceding an increase in the relative humidity from 59.0% to 96.3% (average 80 ± 13.02%), a decrease in temperature from 28.1 °C to 23.2 °C (average 26.2 ± 1.35 °C), and a reduction in rainfall variation, which reached a range of 0.0 to 426.2 mm (average 143.0 ± 148 mm). It is worth noting that during the dry season (August–September 2021 and June 2022), the collection was unsuccessful, or the sandfly activity was practically nil, respectively, demonstrating a strong dependence on the humidity conditions for the development and survival of these insects. In fact, concerning all the species collected in Pedro II, the number of sandflies showed an upward trend during both dry and rainy periods in conditions of high relative humidity, around 70% (Figure 2).

In Teresina, the seasonal pattern was even more pronounced. The highest abundance of *Lu. longipalpis* was observed in the month corresponding to the beginning of the dry season and in the month representing the transition period, especially in June (847/3878) and October (836/3878), respectively. This behavior suggests that, unlike Pedro II in Teresina, the sandfly population responds not only to lower humidity but also to other factors, possibly related to the availability of shelters and urban microenvironments. Significant rainfall was observed during the rainy season; February (600.0 mm) and March (514.2 mm) were the months with the highest concentration of rain, and a consequent increase in the relative humidity, reaching 90%, was observed between January and February. The density of sandflies, mainly *Lu. longipalpis*, remained high during the dry period, indicating greater resilience to low humidity in the urban area of Teresina.

The data show that the population dynamics of sandflies in both locations are closely linked to seasonal variations, particularly in terms of relative humidity. The graphs’ analysis reveals distinct patterns of sandfly abundance in relation to climatic variables, showing that while seasonality in Pedro II is strongly influenced by humidity and the rainy season, in Teresina, there is a greater population persistence, mainly of *Lu. longipalpis* throughout the year, due to less variation in humidity (especially in the dry season) and temperature (Figure 2).

The results of Spearman’s correlation analysis carried out between sandfly vectors and climate variables are shown in Table 2. Significant correlations were found between *Ny. whitmani* collected in Teresina and the following variables: rainfall (ρ = −0.78; *p*-value < 0.01), relative humidity (ρ = −0.80; *p*-value < 0.02), and temperature (ρ = 0.63; *p*-value = 0.02) (Table 2). In Pedro II, a significant result was observed between *Ny. whitmani* and relative humidity (ρ = −0.52; *p*-value = 0.03). In addition, the correlation analysis between climatic factors and the three species of sandflies involved in the transmission cycles of CL in Pedro II (*Ny. whitmani*, *Ny. intermedia,* and *Mi. migonei*) revealed a significant positive correlation between these vectors and rainfall (ρ = 0.58; *p*-value = 0.02) (Table 2).

The relationship between climatic factors and the density of sandflies in Oeiras and Altos was not analyzed since the captures were only made in one semester, not generating enough data to carry out the analysis.

### 3.3. Relationship Between Altitude and Species of Sandflies

**Figure 3:**
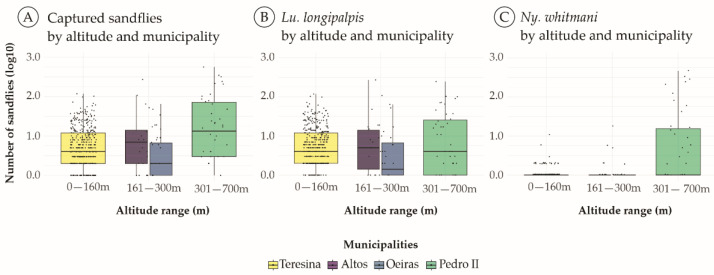
Boxplot representation of the total distribution of sandfly (**A**), *Lu. longipalpis* (**B**), and *Ny. whitmani* (**C**) distributions, in relation to the altitude ranges in the four municipalities of Piauí state. m; meters. Black dots represent individual observations, that is, the actual collection value at a sampling point.

It was observed that altitude had a fundamental impact on defining the distribution patterns of the species of sandflies under study. The analysis of altitude revealed wide variation, from 59 to 700 m with an average of 134.25 ± 130.3 m, favoring a great diversity of phlebotomine species along different altitudes. The box plot illustrating the total count of sandflies revealed a marked distribution of these insects in the altitude range of 301–700 m (green box), representing the municipality of Pedro II (Figure 3A). The distribution of *Lu. longipalpis* showed consistent patterns, with prominent occurrence in all altitude ranges in the four municipalities, with a moderate presence in the altitude interval of 301 to 700 m (green box) compared to the lowest altitudes of Teresina (0–160 m). It is worth noting that in Pedro II, even though the total number of *Lu. longipalpis* was lower, the median and interquartile range (Q1 to Q3) of these individuals per collection point were higher when compared to Teresina. This means that, on average, each collection point in Pedro II had more *Lu. longipalpis* individuals, while in Teresina, despite a greater number of collection sites, fewer individuals were collected in each of these points, resulting in a dilution of the median, which leads to a flattening of the box (yellow box) (Figure 3B). For *Ny. whitmani*, its abundance revealed a remarkable clustering of individuals in Pedro II, with very low distribution in the other municipalities, demonstrating a selective dispersal of this species (Figure 3C).

The correlation between *Lu. longipalpis*, *Ny. whitmani*, and altitude, revealed a significant positive association (ρ = 0.22, *p*-value < 0.01) between the abundance of *Ny. whitmani* and altitude, indicating a preference of this species for higher altitudes. In addition, a significant positive correlation was observed between total sandfly count and altitude (ρ = 0.76, *p*-value = 0.02) (Table 3).

### 3.4. Ecological Analysis of the Altitudinal, Thermal, and Vector Distribution of Sandflies

**Figure 4:**
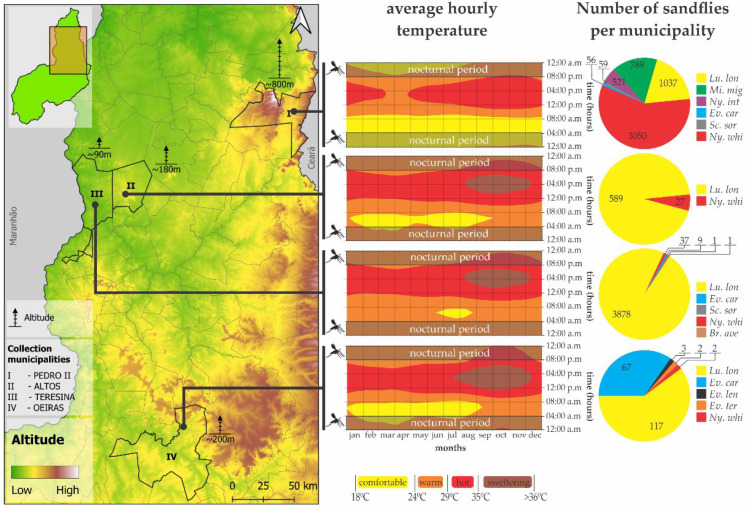
Relationship between the altitude and temperature of each municipality and the presence of phlebotomine sandflies. The map shows the locations of the four municipalities with their respective altitudes, followed on the right by a heat graph showing the hourly/monthly temperature ranges with emphasis on the nocturnal thermal regime of each city. Black insects on the left represent the activity range corresponding to the night period, and the pie charts indicate the faunal density for each sandfly species per collection municipality. Lu. long: *Lu. longipalpis*; Ev. carm: *Ev. carmelinoi*; Ev. lent: *Ev. lenti*; Ev. termit: *Ev. termitophila*; Ny. whit: *Ny. whitmani*; Ny. int: *Ny. intermedia*; Sc. sor: *Sc. sordellii*; Mi. mig: *Mi. migonei.*

The analysis in Figure 4 shows how abiotic factors, such as altitude and average monthly nighttime temperature, directly influence the composition and abundance of sandflies in the different municipalities.

In the highest city of Pedro II (~800 m), while the most comfortable nocturnal thermal period occurs between January and August (8:00 p.m. to 12:00 a.m.), the period between 12:00 a.m. and 4:00 a.m. presents a comfortable thermal spectrum during all months of the year, with a predominance of thermal ranges classified as hot and warm during the daytime period between 4:00 a.m. and 8:00 p.m. These conditions favor both the presence of wild species and of those adapted to more preserved and milder environments, such as *Ny. whitmani* (3050/3116), which was the most abundant species in this municipality. In addition, vector diversity was substantially higher, with a significant presence of *Lu. longipalpis*, *Mi. migonei*, and *Ny. intermedia*, beyond fewer specimens of *Ev. carmelinoi* and *Sc. sordellii*.

On the other hand, in lower altitude areas, such as Teresina (~90 m), Altos (~180 m), and Oeiras (~200 m), warmer and drier conditions prevail at night, with long periods classified as hot and sweltering. These characteristics clearly favor the proliferation of *Lu. longipalpis*, a vector that is highly adapted to anthropized and urban environments. In Teresina, for example, this species accounted for practically all the phlebotomine sandfly fauna recorded, indicating a strong ecological dominance in urban settings with low thermal variation (Figure 4).

### 3.5. Detection of Leishmania *spp.* DNA in Female Sandflies

The presence of *Leishmania* spp. DNA in sandflies was analyzed in 838 non-engorged females divided into 86 groups or pools, each one composed of a maximum of 10 individuals. Of the total number of females analyzed, 425 were *Lu. longipalpis*, 387 *Ny. whitmani*, and 26 *Ny. intermedia*. Minimum positivity was observed in 0.47% of *Lu. longipalpis* specimens (2/425) and 0.25% of *Ny. whitmani* (1/387); that is, two positive pools for *Lu. longipalpis* with at least one individual each presenting *Leishmania* DNA, and one *Ny. whitmani* positive pool containing at least one individual with the presence of parasite DNA. The presence of *Leishmania* DNA was not detected in *Ny. intermedia*.

DNA samples from the three positive pools were later analyzed by hsp70-PCR, cloned, and submitted to sequencing to identify the species of *Leishmania*. According to the BLAST analysis (Table 4), it was possible to reveal the presence of *L. braziliensis* DNA in one pool constituted by nine *Lu. longipalpis* individuals captured in an urban area of Teresina (100% identity) and in another pool of the same species, composed of seven individuals from a rural zone of Oeiras, that presented 98.3% identity with the hsp70 gene sequence of *L. braziliensis*. Curiously, the unique positive pool formed by 10 *Ny. whitmani* individuals collected in a rural zone of Pedro II revealed the presence of DNA of both *L. braziliensis* (100% identity) and *L. infantum* (98.6%) (Table 4). This result suggests, at least, one solely *Ny. whitmani* specimen containing DNAs from the two *Leishmania* parasites (mixed DNAs) or the minimum presence of two individuals, each containing DNA from a single *Leishmania* sp. (Table 4).

### 3.6. Analysis of Blood Sources in Engorged Female Sandflies

Female sandflies (n = 99) of three species, *Lu. longipalpis*, *Ny. whitmani* and *Ny. intermedia*, were identified with traces of blood. In 71 of the 99 samples (71.72%), it was possible to identify four vertebrate species as food sources, human blood being the main source of feeding (52/71, 73.24%). Due to the lack of similarity with sequences available in the database or the poor quality of sequences, it was not possible to identify the food source of 28 samples analyzed (Table S2).

**Figure 5:**
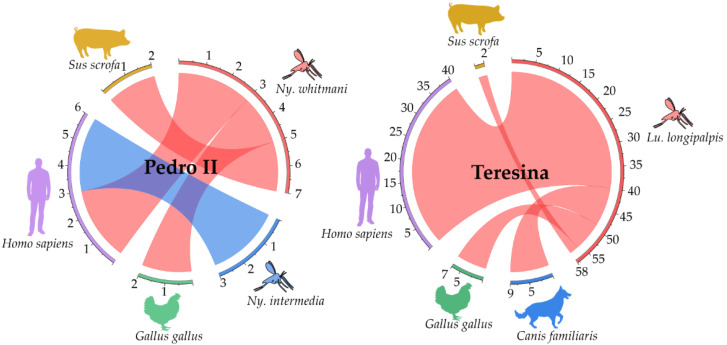
Chord diagram showing the flow of vertebrate species identified in the blood of sandflies, by municipality. Pedro II: 10 female sandflies of the species *Ny. whitmani* and *Ny. intermedia* (right half of the circle) were found engorged with pig (n = 1), human (n = 6), and chicken (n = 2) blood. Teresina: 58 *Lu. longipalpis* females (right half of the circle) were fed on four different vertebrates, most of them (40/58) being identified as human blood. There was no significant difference in sandfly feeding patterns between the studied municipalities (χ^2^ = 7.584, df = 3, *p* = 0.056).

In Teresina, sequencing results of the 12S rRNA gene amplified products related to 58 *Lu. longipalpis* individuals yielded the following distribution of food sources. Almost 69.00% (40/58) fed on *Homo sapiens*, 15.52% (9/58) were identified with *Canis familiaris* blood, 12.07% (7/58) with *Gallus gallus*, and in 3.45% (2/58) *Sus scrofa* blood was detected (Figure 5) (Table S2). In Pedro II, 10 individual samples were analyzed, 7 *Ny. whitmani* and 3 *Ny. intermedia*, of which 60.00% (6/10) had sequences corresponding to *Homo sapiens* (3 *Ny. whitmani*, 3 *Ny. intermedia*) and 20.00% each were positive *for Gallus gallus* and *Sus scrofa*, representing 4 *Ny. whitmani* individuals (Figure 5) (Table S2). Beyond Teresina, 3 *Lu. longipalpis* females were identified containing *Homo sapiens* blood, one collected in Altos and two in Oeiras.

**Figure 6:**
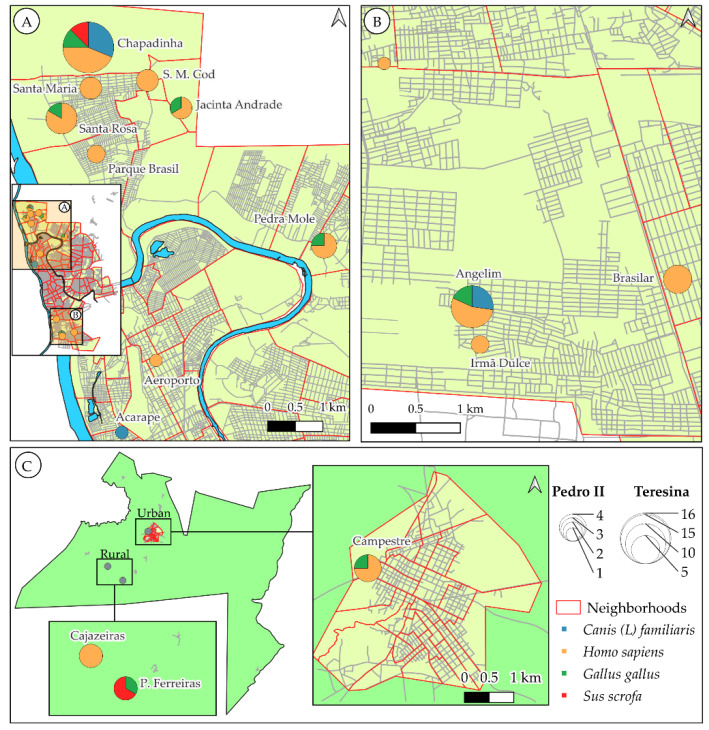
Distribution map of the intestinal blood content analysis of sandflies collected in Teresina and Pedro II. (**A**) North zone, part of the east zone and the center of Teresina; S. M. Cod.: Santa Maria da Codípe; (**B**) South zone of Teresina; (**C**) Urban and rural zones of Pedro II.

Concerning the geographic distribution of engorged sandflies relative to their blood meal sources, we found in Teresina that *Lu. longipalpis* fed on humans in all collection sites. Chapadinha, the most rural area of the northern zone, represented the collection site with the greatest diversity of food sources, including all four vertebrates, with emphasis on canine and human blood. In the peripheral rural areas (Santa Maria, Santa Maria da Codípe-S. M. Cod., Jacinta Andrade, Santa Rosa, Parque Brasil) and in peri-urban regions of Teresina (Pedra Mole, Angélica, Brasilar, Angelim, Irmã Dulce), human blood predominated. Chicken blood has also been recorded in some locations, while sandflies feeding on dogs were observed in Angelim (Figure 6).

In the urban area of Pedro II, specifically in the Campestre neighborhood, human and chicken blood were identified. Concerning the rural zone, sandflies fed exclusively on human blood (Cajazeiras), while in ‘Palmeira dos Ferreira’, chicken and pig were detected (Figure 6).

Figure 6 shows different patterns of the spatial analysis of blood sources between the two municipalities, represented by pie charts superimposed on the neighborhoods where the collections were carried out (Figure 6) (Table S2).

## 4. Discussion

Sandflies play a central role in the transmission of the etiological agents of leishmaniasis, as it is in the digestive tract of the insect vector that Leishmania spp. assumes its infectious metacyclic form, with a species-specific relationship between the parasite and the vector [1,4,5]. In this context, the transmission profile and environmental characteristics are very important in the process of balancing the elements involved in the epidemiological triad of the disease. Our study, conducted in four municipalities in the state of Piauí, provides valuable information on the diversity of sandfly species and their relationship with transmission cycles, demonstrating the occurrence of cross-infection between vectors and parasites from different cycles.

The analysis of the faunal composition of sandflies showed considerable diversity among municipalities, with *Lu. longipalpis* and *Ny. whitmani* standing out and accounting for more than 85% of the total number of collected individuals. The predominance of both species is consistent with other studies carried out in urban and rural areas of Brazil, where *Lu. longipalpis* is often associated with anthropogenic environments [8,10,19,46], while *Ny. whitmani* has been widely recorded in regions with more preserved vegetation and high-altitude environments [17,47,48]. This differentiated distribution among species reflects the influence of the degree of urbanization on vector composition and may indicate the coexistence of different *Leishmania* transmission cycles in the state of Piauí.

The analysis of climatic variables on sandfly density in Teresina and Pedro II revealed contradictory seasonal trends. Although the literature shows the preference of *Ny. whitmani* for milder climates [17,49,50], our results demonstrated a peak of abundance during the dry season (n = 1650), in July 2021, and lesser but still considerable abundance during the rainy period, from December 2021 to February 2022, in Pedro II. In Teresina, this species was almost never found, mainly due to the higher temperature compared to Pedro II. The species *Lu. longipalpis* maintained an irregular presence in Pedro II and was consistently collected throughout the year in Teresina, with a slight increase in the number of individuals recorded during the dry season (May to October 2021), with humidity varying between 70 and 80%. Previous studies identified humidity as a crucial component for larval growth and phlebotomine sandfly activity [50,51,52,53]. The constant presence of *Lu. longipalpis* in urban environments, even in a less-than-ideal humidity condition, indicates a beneficial ecological adaptation that could prolong the risk period for *L. infantum* transmission in densely populated regions.

Seasonal variations can also influence reproductive behavior and interactions of these vectors, including but not limited to variations in temperature and relative humidity [52]. According to Ward (1993) and Kelly (1997) [53,54], sandflies’ pheromones, often influenced by climate, can induce the birth of male individuals, considering that females take more time to emerge from the pupa. This scenario illustrates what we have observed in our study: proportions of ~74% and ~25% for males and females, respectively, bringing together all municipalities of the collection.

Considering the altitude, the results presented here are in agreement with previous reports highlighting the fundamental influence of this environmental variable on the distribution patterns of sandfly species [55,56,57]. The spatial total distribution of species in the altitudinal range of 401–700 m, in Pedro II, shows the influence of high altitudes on the ecology of these vectors, which can be attributed to other factors, including temperature, humidity, and vegetation [47,51,58]. These variables represent microclimate characteristics of higher altitudes, which offer appropriate refuges and favorable conditions for the reproduction and persistence of certain species. The distribution pattern of *Ny. whitmani* clearly showed its predominance in the highest-altitude city, Pedro II, demonstrating a significant positive correlation between species density and altitude. On the other hand, the dispersion of a smaller number of *Ny. whitmani* individuals at lower altitudes shows their developmental ability in environments with considerably lower elevations, possibly due to attenuated temperature variations. It is important to emphasize that the finding of a few *Ny. whitmani* specimens in Teresina is probably favored by the presence of microenvironments that allow for the presence of this species in small populations.

*Lu. longipalpis*, compared to *Ny. whitmani*, which exhibits a more restricted distribution pattern influenced by altitude, revealed wide dispersion at different altitude levels, showing its considerable ecological plasticity [19,59]. While it is frequently found in lower-lying areas, the species is not strictly limited to them, and its presence can extend to higher altitudes, particularly in certain regions. The species *Lu. longipalpis* is regularly associated with lower elevations (<450 m) and specific geoclimatic characteristics like dry climates [47].

In general, our findings confirm previous studies emphasizing the ability of *Ny. whitmani* to adapt to transitional regions between forests and ecotones, while *Lu. longipalpis* demonstrates broad adaptation to urban and peri-urban contexts, as already observed [17,59,60,61]. Understanding the complex interplay between altitude, climate, and other environmental factors is crucial for predicting and managing the spread of leishmaniasis [17].

The high prevalence of *Lu. longipalpis* may facilitate the interaction between the vector and parasites of different transmission chains. In a recent study of our group carried out between 2019 and 2021 in an urban area with remaining rural activity in Altos, Piauí, almost 99% of the collected sandflies were identified as *Lu. longipalpis*. Retrospective data (2008–2018) on the confirmed cases of leishmaniasis in Piauí revealed that this urban area concentrated almost 54% of cutaneous and 86.8% of visceral leishmaniasis cases in the state. We were able to identify one *Lu. longipalpis* individual positive for the presence of both *L. infantum* and *L. braziliensis* DNAs [46]. Here, we confirmed the detection of *L. braziliensis* DNA in *Lu. longipalpis* collected in both urban (Teresina) and rural (Oeiras) areas of Piauí. These data are of great importance and challenge the traditional concept of vector-parasite specificity. Although this sandfly species is classically recognized as a vector of *L. infantum*, other studies have suggested the possibility of infection by other *Leishmania* parasites causing the cutaneous clinical form of the disease. For instance, the report of *Lu. longipalpis* containing *Leishmania amazonensis* DNA in Caxias, a county of Maranhão, a state neighboring Piauí in Northeastern Brazil [5].

In the present study, even more remarkable was the simultaneous detection of *L. braziliensis* and *L. infantum* DNAs in a solely pool formed by *Ny. whitmani* specimens, suggesting the possibility of mixed infection in one or more individuals or the presence of at least two individuals, each carrying genetic material of a different parasite species. It should be noted that working with pools does not clarify the probability of mixed infection, which could clearly be resolved through individual analyses of collected specimens, attributing the infection to a single specimen. Nevertheless, these findings indicate greater vector interactivity than previously recognized and may have direct epidemiological implications, especially in the context of overlapping transmission cycles. The detection of *L. infantum* DNA in *Ny. whitmani* in Pedro II suggests a possible adaptation of the vector in the transmission of the causative agent of VL in the northern region of Piauí. This finding indicates, at least, closer contact of both vector and parasite, which in turn can be implicated in the competence and vectorial capacity of *Ny. whitmani* to sustain the cycle of *L. infantum*. It is also important to consider the three times higher density of this vector in relation to *Lu. longipalpis* in Pedro II (a condition not expected in other municipalities of Piauí), which could narrow the proximity between *Ny. whitmani* and *L. infantum* during the blood meals on infected hosts.

The species *Ny. whitmani* has been widely studied for its role in the transmission of the main causative agent of CL in several regions of Brazil [60,61], as well as *Nyssomyia shawi* in the state of Acre [62]. Our data corroborate emerging evidence suggesting that *Ny. whitmani* may act as a potential vector of *L. infantum* in rural areas of Argentina with confirmed endemicity for this parasite species [48,63]. This possible association has gained prominence due to the detection of other sandfly species (*Pintomyia fischeri*, *Mi. migonei,* and *Ny. neivai*) parasitized with *L. infantum* in VL endemic areas, where the presence of the main vector of this parasite, such as *Lu. longipalpis* in the New World, is limited or nonexistent [47,64,65,66,67].

In our work, a similar situation was observed in Pedro II, where *Ny. whitmani* was collected in greater quantities compared to *Lu. longipalpis*, as mentioned above. The association between *Ny. whitmani* and *L. infantum* has particular significance in geographical areas where the prevalence of VL has had a notable expansion, thus representing a substantial public health concern. A comprehensive analysis of the vector competence of *Ny. whitmani*, specifically with regard to its ability to acquire, maintain, and transmit *L. infantum*, is crucial to elucidate its true role in transmitting this parasite [48].

These findings, for both *Lu. longipalpis* and *Ny. whitmani*, suggest the occurrence of continuous adaptive changes that enable the vectors to explore new habitats, thus potentially expanding the spread of parasites that are not part of their inherent transmission cycles. Similar results have been found in other studies, demonstrating that the dispersion of phlebotomine sandflies does not restrict a vector species to a single etiological agent [8,49,50,63]. Unplanned urbanization and changes in the environment are elements that promote greater interactions between vectors, reservoirs, and human populations [19,68], creating an advantageous scenario for the emergence of a ‘new’ ecoepidemiological panorama of leishmaniasis.

Blood content analyses of engorged females revealed different feeding patterns among species and municipalities, taking into consideration the few engorged females analyzed from Pedro II compared to the greater number collected in Teresina. While *Ny. whitmani* and *Lu. longipalpis* demonstrated a more varied diet, the same was not observed for the three *Ny. intermedia* individuals that fed exclusively on humans. In our study, we observed a high rate of sandflies feeding on humans (~73%); the majority referred to *Lu. longipalpis* females widely distributed in Teresina. The high anthropophilia of this sandfly was previously described, bringing an alert for the risk of *L. infantum* transmission in densely populated areas [68].

A greater diversity of blood meal sources was seen in rural areas such as ‘Chapadinha’, in the northern zone of Teresina, and ‘Palmeira dos Ferreiras’, in Pedro II. This probably occurs due to the consistent coexistence of several domestic animals, including chickens, pigs, dogs, and others, maintained in shelters around the houses. In these specific ecological habitats, both the resident fauna and the human population itself serve as persistent attractants for sandflies, highlighting the importance of peridomiciliary transmission of *Leishmania* spp., as shown in other studies [60,68,69,70]. Dogs have been implicated as the main domestic reservoirs of *L. infantum*, and their close association with humans facilitates parasite transmission by sandflies to humans. The presence of infected dogs in an area directly influences the risk of transmission and the persistence of VL, making this vertebrate host a critical component in peridomestic and domestic transmission foci [71].

In the rural zone of Pedro II, we observed *Ny. whitmani* feeding exclusively on humans (‘Cajazeiras’) or on pigs and chickens (‘Palmeira dos Ferreiras’), suggesting a less selective feeding behavior of this sandfly, able to explore human and non-human hosts available in the environment. The diversity of hosts found in the peridomicile has an influence on the transmission cycle, as these vertebrates can act as both a barrier and an amplifier of the parasite transmission, depending on the vector’s competence and the parasite load of reservoirs. Previous studies have shown that sandflies need a second blood supply to increase vector infectiousness by promoting *Leishmania* replication via a novel developmental stage, the retroleptomonads. This second supply is linked to a possible ‘preference’ of vector females for chicken blood, making these birds a readily available blood source for phlebotomine sandflies with an indirect role in the transmission cycle [72].

It is well documented that the geographic spread and urbanization of VL in Brazil have occurred in recent decades. This scenario can be related to population migration, environmental destruction, facilitating the direct contact between humans, natural vector breeding habitats, and wild reservoirs, or disorderly land occupation with poor sanitation that favors the vector’s adaptation to the peridomestic environment [19]. A rapid uncontrolled urban expansion to adjacent rural areas where zoonotic cycles of leishmaniasis occur contributes to the rapid propagation of the disease and an increased risk of human infection [73]. Considering this panorama, it is important to improve entomological surveillance efforts and implement effective vector control strategies in the affected regions.

The identification of vectors presenting *Leishmania* DNA of other species that are not part of their inherent cycle of transmission, and the acclimatization of pre-existing species to urban areas, reinforce the need for more comprehensive and cohesive approaches for vector control in the studied municipalities. Strategies for vector vigilance should include environmental sanitation measures, health education initiatives, and proper management of human and canine cases.

The present work has some limitations, such as: (i) The sampling duration was not uniform across the study areas, reducing seasonal comparability with the exclusion of the municipalities of Oeiras and Altos, which could not be monitored continuously, and likely affecting the interpretation of vector distribution under the interference of climatic variables in these areas. (ii) Molecular analysis of the presence of both *L. braziliensis* and *L. infantum* DNAs in a pool of 10 *Ny. whitmani* specimens did not clarify whether co-infection (mixed DNAs) occurred in the same vector or if different individuals were infected; indeed, working with insect pools, we can only infer minimal infection positivity. (iii) The sample size of engorged females was small; therefore, the results of food source identification cannot accurately represent the actual situation of host diversity in rural and urban areas of Teresina and Pedro II.

## 5. Conclusions

A faunal survey in four municipalities of Piauí revealed the predominance of *Lu. longipalpis*, the species most widely distributed in urban and rural areas, corroborating its notorious capacity to adapt to anthropized environments, such as Teresina, the capital of the state, a densely populated city. Pedro II, a county with the highest altitude and mild climate, exhibited a greater number of *Ny. whitmani* individuals compared to *Lu. longipalpis*. In the correlation analyses between vector density and climate variables, *Ny. whitmani* revealed significant results, reflecting its seasonal behavior controlled by temperature, humidity, and rainfall in Teresina, and by the humidity variable in Pedro II. It is worth emphasizing that climatic analyses were only valid for continuously monitored areas, leading to the exclusion of Oeiras and Altos, where collections were conducted in a minimum period of 3–6 months.

The detection of at least two *Lu. longipalpis* individuals (from an urban area of Teresina and the rural zone of Oeiras) harboring *L. braziliensis* DNA and the finding of *L. infantum* and *L. braziliensis* DNAs in a solely pool formed by *Ny. whitmani* from Pedro II draw attention to the possible emergence of ‘cross-infection’ with other vectors participating in the epidemiology of cutaneous and visceral leishmaniasis in the state of Piauí. However, experimental infection studies are needed to formally establish the competence of these sandflies. The search for the feeding habits of sandflies identified humans as the main blood source, reinforcing the importance of entomological surveillance for understanding the transmission dynamics of *Leishmania* parasites in the studied areas.

## Figures and Tables

**Table 1:** Abundance and distribution of sandflies collected from November 2020 to August 2022 in four municipalities in the state of Piauí, Brazil.

Species	Teresina				Altos		Pedro II			Oeiras		Total Number	Abundance (%)
	Intra	Peri	T.Tr	Total	Peri	Total	Intra	Peri	Total	Peri	Total		
*Lu. longipalpis*	527	3281	70	3878	589	589	135	902	1037	117	117	5621	54.87
*Ny. whitmani*	17	20		37	27	27	228	2822	3050	2	2	3116	30.41
*Mi. migonei*								789	789			789	7.70
*Ny. intermedia*							2	519	521			521	5.09
*Ev. carmelinoi*	1	7	1	9			11	45	56	67	67	132	1.29
*Sc. sordellii*	1			1				59	59			60	0.59
*Ev. lenti*										3	3	3	0.03
*Ev. termitophila*										2	2	2	0.02
*Br. avellari*		1		1								1	0.01
TOTAL	546	3309	71	3926 *	616	616	376	5136	5512 *	191	191	10,245	100.00

T.Tr: Top of the tree; Intra: Intradomicile; Peri: Peridomicile. *: comparison of the total number of sandflies collected in the municipalities of Teresina and Pedro II [Kruskal–Wallis test. H = 8.0, degrees of freedom (df) = 8, *p* > 0.05]; abbreviation of genera according to Marcondes et al. in 2007 [36].

**Table 2:** Spearman’s correlation coefficients (ρ) between the density of sandfly vectors and climatic variables in the municipalities of Teresina and Pedro II.

Municipalities	Sandflies	Climate Data		
		Rainfall—mm (*p*-Value)	Temp—°C (*p*-Value)	R.H—% (*p*-Value)
Teresina	*Lu. longipalpis*	−0.39 (0.25)	0.35 (0.26)	−0.45 (0.13)
	*Ny. whitmani*	−0.78 (<0.01 *)	0.63 (0.02 *)	−0.80 (<0.02 *)
	Total sandflies	−0.36 (0.25)	0.35 (0.26)	−0.45 (0.26)
Pedro II	*Lu. longipalpis*	0.17 (0.55)	−0.14 (0.62)	−0.34 (0.23)
	*Ny. whitmani*	0.11 (0.71)	0.26 (0.36)	−0.52 (0.03 *)
	*Ny. intermedia*	0.41 (0.14)	−0.31 (0.27)	−0.12 (0.68)
	*Mi. migonei*	0.26 (0.36)	−0.17 (0.55)	−0.03 (0.90)
	ACL Vectors	0.58 (0.02 *)	−0.02 (0.95)	−0.17 (0.55)
	Total sandflies	0.42 (0.13)	0.06 (0.82)	−0.32 (0.90)

ACL: American Cutaneous Leishmaniasis; Temp: Temperature; R.H: Relative Humidity; Bold *: Significant *p*-Value (*p* < 0.05).

**Table 3:** Spearman’s correlation coefficients (ρ) of the analysis between *Nyssomyia whitmani* and *Lutzomyia longipalpis* species and the altitude of the studied municipalities in Piauí.

Species	Correlated Variable	Correlation (ρ)	*p*-Value
*Lu. longipalpis*	Altitude	0.03	0.36
*Ny. whitmani*	Altitude	0.22	<0.01 *
Total of both species	Altitude	0.76	0.02 *

* *p*-value < 0.05.

**Table 4:** Species of phlebotomine sandflies that tested positive for *Leishmania* spp. kDNA according to the municipality, collection zone, and the species of *Leishmania* identified.

Mun.	Zone	N/L	Sandfly	Parasite sp. (hsp70)	Accession Number	BLAST Analysis		Positive Pool/Insects
						E-V	ID (%)	
THE	Urb	Angelim	*Lu. longipalpis*	*L.* (*V*.) *braziliensis*	MN395479.1	2E-51	100.0	1/9
OEI	Rur	Queiroz	*Lu. longipalpis*	*L.* (*V*.) *braziliensis*	MN395479.1	9E-49	98.3	1/7
PII	Rur	P. Ferreiras	*Ny. whitmani*	*L.* (*V*.) *braziliensis*	MN395479.1	4E-67	100.0	1/10
				*L*. (*L*.) *infantum*	MF137828.1	3E-63	98.6	

Mun.: Municipality; THE: Teresina; OEI: Oeiras; PII: Pedro II; Urb: Urban; Rur: Rural; N/L: Neighborhood/Location; P. Ferreiras: Palmeira dos Ferreiras; hsp70: Target Gene for the Identification of *Leishmania* sp.; E-V: E-Value; ID: Identity.

## Data Availability

The original contributions presented in this study are included in the article/Supplementary Material. Further inquiries can be directed to the corresponding author.

## Supplementary Materials

The following supporting information can be downloaded at https://www.mdpi.com/article/10.3390/pathogens14090930/s1: Table S1: Sandfly captures’ strategy from November 2020 to August 2022 in four municipalities in the state of Piauí, Brazil; Table S2: Consolidated analysis of blood gut-contents in sandflies in the municipalities of capture; Figure S1: Heat map graph showing the temperature overview for 12 months of the year in all collection municipalities. Data source: https://pt.weatherspark.com/ (accessed on 23 May 2023).

## Abbreviations

The following abbreviations are used in this manuscript:

VL	Visceral Leishmaniasis
ACL	American Cutaneous Leishmaniasis
L	*Leishmania*
Lu.	*Lutzomyia*
Ny.	*Nyssomyia*
Mi.	*Migonemyia*
Ev.	*Evandromyia*
Sc.	*Sciopemyia*
Br.	*Brumptomyia*
