# Supplementary material for: Ecological Perspectives on Leishmaniasis Parasitism Patterns: Evidence of Possible Alternative Vectors for Leishmania (Leishmania) infantum (syn. L. chagasi) and Leishmania (Viannia) braziliensis in Piauí, Brazil
Source: Pathogens. 2025 Sep 16;14(9):930. doi: 10.3390/pathogens14090930 (PMC12472939; doi:10.3390/pathogens14090930)
Supplement: Supplementary file 1 [file pathogens-14-00930-s001.zip › Table S1.pdf]

Table S1: Sandfly captures' strategy from November 2020 to August 2022 in four municipalities in the state of Piauí, Brazil

Municipalities	Capture	Number of HP/CDC light traps					Operating time (OT)	Number of nights in operation (NN)	Sampling effort HP traps (TT x OT x NN)
		Intra (1m-1,90m)	Peri: Ground (1m-1,90m)	Peri: Top of trees (10m)	Total traps	Total traps per night (TT)	(hours)	night (17h - 7h)	Time (hours)
Teresina	Rainy season Nov to Dec 2020	2	6		8	4	14h (17h - 7h)	2	112:00 h
	Rainy season Jan to May 2021	7	112	5	124	4	14h (17h - 7h)	31	1,736:00 h
	Dry season Jun to Oct 2021	44	136		180	6	14h (17h – 7h)	30	2,520:00 h
	Rainy season Nov 2021	4	16		20	5	14h (17h – 7h)	4	280:00 h
Altos	Rainy season Jan to May 2021		12		12	4	14h (17h – 7h)	3	168:00 h
	Dry season Jun to Aug 2021		16		16	4	14h (17h – 7h)	4	224:00 h
Oeiras	Rainy season Mar to May 2021		4		4	1	14h (17h – 7h)	4	56:00h
	Dry season Jun to Aug 2021		12		12	4	14h (17h – 7h)	3	168:00 h
Pedro II	Dry season Jun to Oct 2021	6	9		15	5	14h (17h – 7h)	3	210:00 h
	Rainy season Nov to Dec 2021	2	10		12	3	14h (17h – 7h)	4	168:00 h
	Rainy season Jan to May 2022		20		20	2	14h (17h – 7h)	10	280:00 h
	Dry season Jun to Aug 2022		8		8	2	14h (17h – 7h)	4	112:00 h
