# Supplementary material for: Ecological Perspectives on Leishmaniasis Parasitism Patterns: Evidence of Possible Alternative Vectors for Leishmania (Leishmania) infantum (syn. L. chagasi) and Leishmania (Viannia) braziliensis in Piauí, Brazil
Source: Pathogens. 2025 Sep 16;14(9):930. doi: 10.3390/pathogens14090930 (PMC12472939; doi:10.3390/pathogens14090930)
Supplement: Supplementary file 1 [file pathogens-14-00930-s001.zip › Table S2.pdf]

Table S2: Consolidated analysis of blood gut-contents in sandflies in the municipalities of capture.

Mun.	Zone	N/L	Sandflies	Blood meal (*12SrRNA*)	A. N°	BLAST analysis		Total
						E-V	Identity (%)	
Altos	Urb	S. Sebastião	*Lu. longipalpis*	*Homo sapiens*	ON496980.1	3,00E-79	99.41	1
Oeiras	Urb	Oeiras Nova	*Lu. longipalpis*	*Homo sapiens*	ON496980.1	8,00E-80	100.00	1
Oeiras	Urb	Oeiras Nova	*Lu. longipalpis*	*Homo sapiens*	KU131353.1	1,00E-57	93.71	1
Pedro II	Urb	Campestre	*Ny. whitmani*	*Gallus gallus*	MN972460.1	2,00E-60	94.97	1
Pedro II	Rur	P. Ferreiras	*Ny. whitmani*	*Gallus gallus*	MN972460.1	2,00E-75	100.00	1
Pedro II	Urb	Campestre	*Ny. whitmani*	*Homo sapiens*	ON496980.1	7,00E-76	96.69	1
Pedro II	Urb	Campestre	*Ny. whitmani*	*Homo sapiens*	MF621103.1	3,00E-74	97.13	1
Pedro II	Urb	Campestre	*Ny. whitmani*	*Homo sapiens*	ON496980.1	2,00E-30	94.62	1
Pedro II	Rur	Cajazeiras	*Ny. intermedia*	*Homo sapiens*	ON496980.1	7,00E-70	100.00	1
Pedro II	Rur	Cajazeiras	*Ny. intermedia*	*Homo sapiens*	ON496980.1	3,00E-69	99.34	1
Pedro II	Rur	Cajazeiras	*Ny. intermedia*	*Homo sapiens*	MT048574.1	3,00E-73	100.00	1
Pedro II	Rur	P. Ferreiras	*Ny. whitmani*	*Sus scrofa*	MT253545.1	1,00E-46	98.23	1
Pedro II	Rur	P. Ferreiras	*Ny. whitmani*	*Sus scrofa*	MT253545.1	2,00E-75	99.39	1
Teresina	Urb	Angelim I	*Lu. longipalpis*	*Canis (L) familiaris*	KX779930.1	3,00E-74	100.00	1
Teresina	Urb	Angelim	*Lu. longipalpis*	*Canis (L) familiaris*	MN181405.1	2,00E-36	97.50	1
Teresina	Urb	Angelim IV	*Lu. longipalpis*	*Canis (L) familiaris*	MN181405.1	6,00E-51	95.25	1
Teresina	Rur	Chapadinha	*Lu. longipalpis*	*Canis (L) familiaris*	MN181405.1	2,00E-80	98.86	1
Teresina	Rur	Chapadinha	*Lu. longipalpis*	*Canis (L) familiaris*	MW487692.1	4,00E-78	97.74	1
Teresina	Rur	Chapadinha	*Lu. longipalpis*	*Canis (L) familiaris*	MN181405.1	2,00E-81	98.87	1
Teresina	Rur	Chapadinha	*Lu. longipalpis*	*Canis (L) familiaris*	MN181405.1	1,00E-68	96.95	1
Teresina	Rur	Chapadinha	*Lu. longipalpis*	*Canis (L) familiaris*	MN181405.1	3,00E-63	96.13	1
Teresina	Urb	Acarape	*Lu. longipalpis*	*Canis (L) familiaris*	MN181405.1	4,00E-62	97.32	1
Teresina	Urb	Pedra Mole	*Lu. longipalpis*	*Gallus gallus*	MN972460.1	2,00E-80	100.00	1
Teresina	Urb	Angelim	*Lu. longipalpis*	*Gallus gallus*	MN972460.1	5,00E-72	98.15	1
Teresina	Urb	Santa Rosa	*Lu. longipalpis*	*Gallus gallus*	MN972460.1	1,00E-78	99.41	1
Teresina	Urb	J. Andrade	*Lu. longipalpis*	*Gallus gallus*	MN972460.1	3,00E-69	100.00	1
Teresina	Urb	Angelim I	*Lu. longipalpis*	*Gallus gallus*	MN972460.1	4,00E-62	96.69	1
Teresina	Rur	Chapadinha	*Lu. longipalpis*	*Gallus gallus*	MN972460.1	7,00E-81	100.00	1

Teresina	Rur	Chapadinha	*Lu. longipalpis*	*Gallus gallus*	MN972460.1	7,00E-60	95.42	1
Teresina	Urb	Brasilar	*Lu. longipalpis*	*Homo sapiens*	MF621103.1	3,00E-63	96.71	1
Teresina	Urb	Sta. M. Cod.	*Lu. longipalpis*	*Homo sapiens*	KT725998.1	3,00E-44	97.88	1
Teresina	Urb	Sta. M. Cod.	*Lu. longipalpis*	*Homo sapiens*	ON496980.1	1,00E-78	99.41	1
Teresina	Urb	Sta. M. Cod.	*Lu. longipalpis*	*Homo sapiens*	ON496980.1	1,00E-73	97.65	1
Teresina	Urb	Santa Rosa	*Lu. longipalpis*	*Homo sapiens*	MN972460.1	1,00E-58	92.31	1
Teresina	Urb	Santa Rosa	*Lu. longipalpis*	*Homo sapiens*	KP702293.1	4,00E-78	98.83	1
Teresina	Urb	Santa Rosa	*Lu. longipalpis*	*Homo sapiens*	ON496980.1	3,00E-79	99.41	1
Teresina	Urb	Santa Rosa	*Lu. longipalpis*	*Homo sapiens*	ON496980.1	7,00E-81	99.42	1
Teresina	Urb	Santa Rosa	*Lu. longipalpis*	*Homo sapiens*	MT048574.1	4,00E-72	100.00	1
Teresina	Urb	Pedra Mole	*Lu. longipalpis*	*Homo sapiens*	KP702293.1	3,00E-37	93.22	1
Teresina	Urb	Angelim I	*Lu. longipalpis*	*Homo sapiens*	ON496980.1	1,00E-78	100.00	1
Teresina	Urb	J. Andrade	*Lu. longipalpis*	*Homo sapiens*	MT048572.1	2,00E-75	100.00	1
Teresina	Urb	Brasilar	*Lu. longipalpis*	*Homo sapiens*	ON496980.1	1,00E-78	100.00	1
Teresina	Urb	Angelim I	*Lu. longipalpis*	*Homo sapiens*	MT048574.1	3,00E-64	95.71	1
Teresina	Urb	P. Brasil III	*Lu. longipalpis*	*Homo sapiens*	ON496980.1	3,00E-73	99.37	1
Teresina	Urb	P. Brasil III	*Lu. longipalpis*	*Homo sapiens*	ON496980.1	2,00E-71	98.15	1
Teresina	Urb	Santa Maria	*Lu. longipalpis*	*Homo sapiens*	MT048572.1	2,00E-75	100.00	1
Teresina	Urb	Angelim I	*Lu. longipalpis*	*Homo sapiens*	ON496980.1	3,00E-74	99.38	1
Teresina	Urb	Angelim I	*Lu. longipalpis*	*Homo sapiens*	ON496980.1	2,00E-76	100.00	1
Teresina	Urb	Angelim I	*Lu. longipalpis*	*Homo sapiens*	ON496980.1	6,00E-71	98.73	1
Teresina	Urb	Angélica	*Lu. longipalpis*	*Homo sapiens*	MN848587.1	3,00E-69	98.10	1
Teresina	Urb	Brasilar	*Lu. longipalpis*	*Homo sapiens*	ON496980.1	1,00E-78	100.00	1
Teresina	Urb	Angelim I	*Lu. longipalpis*	*Homo sapiens*	ON496980.1	2,00E-76	99.39	1
Teresina	Urb	J. Andrade	*Lu. longipalpis*	*Homo sapiens*	ON496980.1	4,00E-73	98.76	1
Teresina	Urb	Irmã Dulce	*Lu. longipalpis*	*Homo sapiens*	ON496980.1	2,00E-70	97.55	1
Teresina	Urb	Brasilar	*Lu. longipalpis*	*Homo sapiens*	ON496980.1	4,00E-73	98.77	1
Teresina	Urb	Brasilar	*Lu. longipalpis*	*Homo sapiens*	ON496980.1	1,00E-72	98.17	1
Teresina	Urb	Irmã Dulce	*Lu. longipalpis*	*Homo sapiens*	ON496980.1	2,00E-70	99.35	1
Teresina	Rur	Chapadinha	*Lu. longipalpis*	*Homo sapiens*	ON496980.1	3,00E-79	98.84	1

Teresina	Rur	Chapadinha	*Lu. longipalpis*	*Homo sapiens*	ON496980.1	2,00E-80	99.42	1
Teresina	Rur	Chapadinha	*Lu. longipalpis*	*Homo sapiens*	KM281527.1	4,00E-73	97.09	1
Teresina	Rur	Chapadinha	*Lu. longipalpis*	*Homo sapiens*	KU683129.1	1,00E-77	98.26	1
Teresina	Rur	Chapadinha	*Lu. longipalpis*	*Homo sapiens*	MT048566.1	1,00E-48	95.95	1
Teresina	Rur	Chapadinha	*Lu. longipalpis*	*Homo sapiens*	KP702293.1	1,00E-52	99.71	1
Teresina	Rur	Chapadinha	*Lu. longipalpis*	*Homo sapiens*	EU600365.1	9,00E-19	95.34	1
Teresina	Urb	Santa Maria	*Lu. longipalpis*	*Homo sapiens*	MT048569.1	3,00E-74	100.00	1
Teresina	Urb	Aeroporto	*Lu. longipalpis*	*Homo sapiens*	MT048569.1	2,00E-71	98.73	1
Teresina	Urb	Pedra Mole	*Lu. longipalpis*	*Homo sapiens*	ON496980.1	4,00E-73	99.37	1
Teresina	Urb	Santa Maria	*Lu. longipalpis*	*Homo sapiens*	ON496980.1	3,00E-74	99.38	1
Teresina	Urb	Pedra Mole	*Lu. longipalpis*	*Homo sapiens*	ON496980.1	8,00E-70	97.53	1
Teresina	Rur	Chapadinha	*Lu. longipalpis*	*Sus scrofa*	MT253545.1	3,00E-48	95.47	1
Teresina	Rur	Chapadinha	*Lu. longipalpis*	*Sus scrofa*	MT253545.1	6,00E-50	94.81	1

Sandflies (+), Positive sandflies for Leishmania kDNA; Mun, Municipality; P. Ferreiras, Palmeira dos Ferreiras; Sta. M. Cod, Santa Maria da Codipe; J. Andrade, Jacinta Andrade; P. Brasil III, Parque Brasil III; N/L, Neighborhood / Location; A.N°, Accession number; E-V, E-value; Urb, Urban; Rur, Rural
