# Supplementary figures and images for: Ecological Perspectives on Leishmaniasis Parasitism Patterns: Evidence of Possible Alternative Vectors for Leishmania (Leishmania) infantum (syn. L. chagasi) and Leishmania (Viannia) braziliensis in Piauí, Brazil
Source: Pathogens. 2025 Sep 16;14(9):930. doi: 10.3390/pathogens14090930 (PMC12472939; doi:10.3390/pathogens14090930)

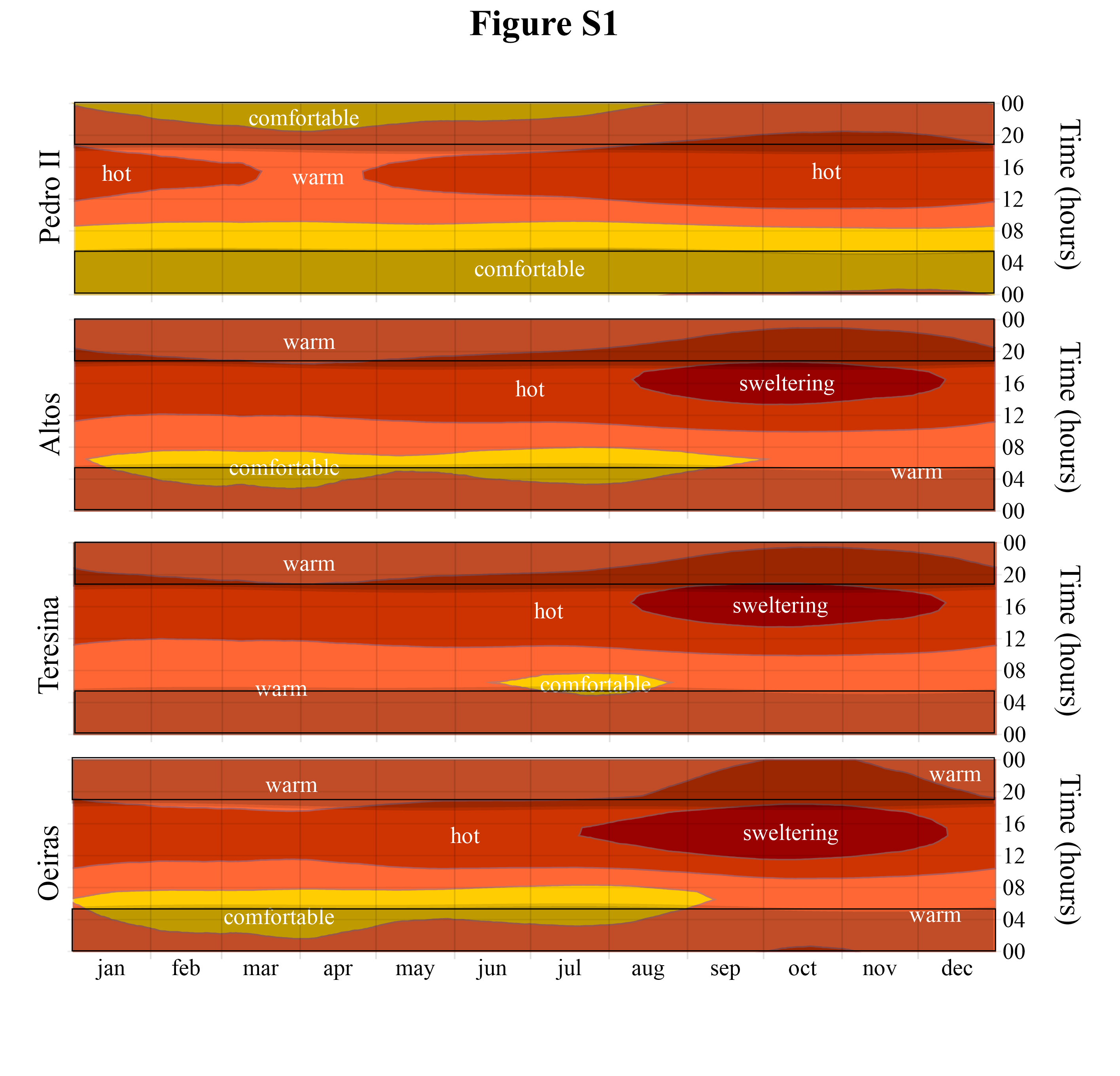

Supplement: Supplementary file 1 [file pathogens-14-00930-s001.zip › Figure S1.tif]
